# The interplay among PbNAC71, PbWAT1, and PbRNF217 reveals the secret behind dwarf pear trees

**DOI:** 10.1093/plphys/kiae035

**Published:** 2024-01-19

**Authors:** Yee-Shan Ku

**Affiliations:** Assistant Features Editor, Plant Physiology, American Society of Plant Biologists; School of Life Sciences and Centre for Soybean Research of the State Key Laboratory of Agrobiotechnology, The Chinese University of Hong Kong, Hong Kong SAR, China

Dwarfism is an important trait for fruit trees. Dwarf fruit trees facilitate fruit picking, minimize pruning cost, and reduce crop waste (Atkinson and Else 2014). To generate dwarf fruit trees, grafting is a common strategy. The roots of dwarf varieties can be used as the rootstocks for grafting to induce dwarfism of the shoots (Atkinson and Else 2014). Breeding could be an alternative to grafting for generating dwarf fruit trees. However, dwarf fruit tree varieties are limited in some species. In addition, the mechanism behind dwarfism has remained unclear in many fruit tree plants. Pear is one of the fruit species that have limited dwarf varieties and unclear dwarfism mechanisms.

In this issue of *Plant Physiology*, Cong et al. reported a novel dwarfism module in pear involving the interplay among PbNAC71, PbWAT1, and PbRNF217, which encode a NAC transcription factor, a secondary wall synthesis-related protein, and an E3 ubiquitin ligase, respectively (Cong et al. 2024). To identify dwarfism-related genes in pear, the authors first generated dwarf pear trees by grafting non-dwarf pear “Zaosu” (Z) shoot with dwarf quince (Q) root (Z/Q). Compared to pear plants generated by grafting non-dwarf pear “Zaosu” (Z) shoot and non-dwarf pear “Duli” (D) root (Z/D), Z/Q plants have reduced plant height, shoot length, and xylem size (Cong et al. 2024). By RNA-sequencing, the authors identified more than 200 differentially expressed genes between Z/Q and Z/D shoots. *PbNAC71* expression was correlated with dwarfism in the crosses and across different pear varieties (Cong et al. 2024). The role of *PbNAC71* in inducing dwarfism was also shown in transgenic tobacco and pear plants. The overexpression of *PbNAC71* in tobacco or pear plants resulted in reduced plant height, plant diameter, xylem size, and vessel area (Cong et al. 2024).

Since the dwarf transgenic plants showed defective xylems, the authors hypothesized that xylem-related genes are regulated by PbNAC71 to result in the dwarfism phenotype. RNA-sequencing showed that the overexpression of *PbNAC71* in pear resulted in the reduced expression of *PbWAT1*. In the model plant *Arabidopsis*, the mutation of *AtWAT1*, the homolog of *PbWAT1*, resulted in dwarfism. The dwarfism phenotype could be rescued by overexpressing *PbWAT1*. The results supported the negative role of *PbWAT1* in dwarfism.

The function of PbNAC71 was also dissected at the molecular level. The binding between *PbWAT1* promoter and PbNAC71 was shown by yeast-one-hybrid, immunoprecipitation-qPCR, and electromobility shift assays. The transcriptional repression of *PbWAT1* promoter by PbNAC71 was revealed by dual luciferase assay. Moreover, a yeast-two-hybrid assay was used to search for protein interacting partners of PbNAC71. The assay suggested the binding between PbNAC71 and PbRNF217. The binding was further verified by bimolecular fluorescence complementation. Since *PbRNF217* encodes an E3 ubiquitin ligase, the authors hypothesized that PbRNF217 targets PbNAC71 for ubiquitination and degradation.

The degradation of PbNAC71 by PbRNF217 mediated ubiquitination was demonstrated using transgenic systems. The co-expression of *PbRNF217* with *PbNAC71* in transgenic tobacco promoted the ubiquitination of PbNAC1. The co-expression in transgenic pear root also reduced the accumulation of PbNAC1. In addition, the dwarfism phenotype resulted from *PbNAC1* overexpression could be partially rescued by *PbRNF217* co-expressed.

Altogether, the results suggested that PbNAC71 plays a positive role in dwarfism by repressing the expression of *PbWAT1*. Moreover, the accumulation of PbNAC71 is regulated by PbNRF217, which mediates the ubiquitination of PbNAC71 for degradation. The interplay among PbNAC71, PbWAT1, and PbNRF217 is summarized in Fig. Despite the popularity of using dwarfing rootstocks for grafting, the mechanism behind the scion dwarfism has remained unclear. This study by Cong et al. in pear showed that the dwarfing rootstock effects the expression of *PbNAC71* in the scion and revealed the dwarfism mechanism regulated by the interplay among PbNAC71, PbWAT1, and PbNRF217 (Cong et al. 2024). In addition to explaining the scion dwarfing mechanism, this study also facilitates molecular breeding for dwarf pear varieties and provides a reference for woody plants.

**Figure.**
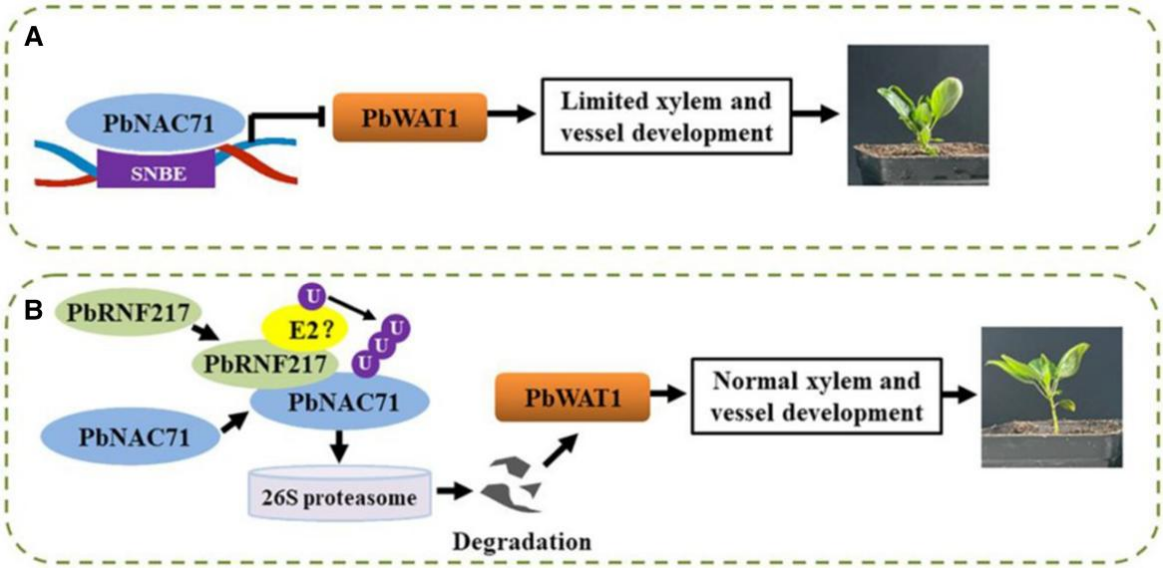
The interplay among PbNAC71, PbWAT1, and PbRNF217 regulates dwarfism in pear. **A)** PbNAC71 binds to the secondary wall NAC binding element (SNBE) in the promoter of *PbWAT1* to repress its expression. Such a transcriptional repression results in limited xylem and vessel development, and thus the dwarf phenotype. **B)** PbNRF217 targets PbNAC1 for ubiquitination (U)-mediated degradation by the 26S proteasome. The degradation abolishes the transcriptional repression of *PbWAT1* to result in normal xylem and vessel development, and the non-dwarf phenotype. This figure is adapted from Cong et al. 2024.

## Dive Curated Terms

The following phenotypic, genotypic, and functional terms are of significance to the work described in this paper:

NAC CHEBI: CHEBI:7421
